# Supplementary figures and images for: Person-to-person opinion dynamics: An empirical study using an online game
Source: PLoS One. 2022 Oct 6;17(10):e0275473. doi: 10.1371/journal.pone.0275473 (PMC9536623; doi:10.1371/journal.pone.0275473)

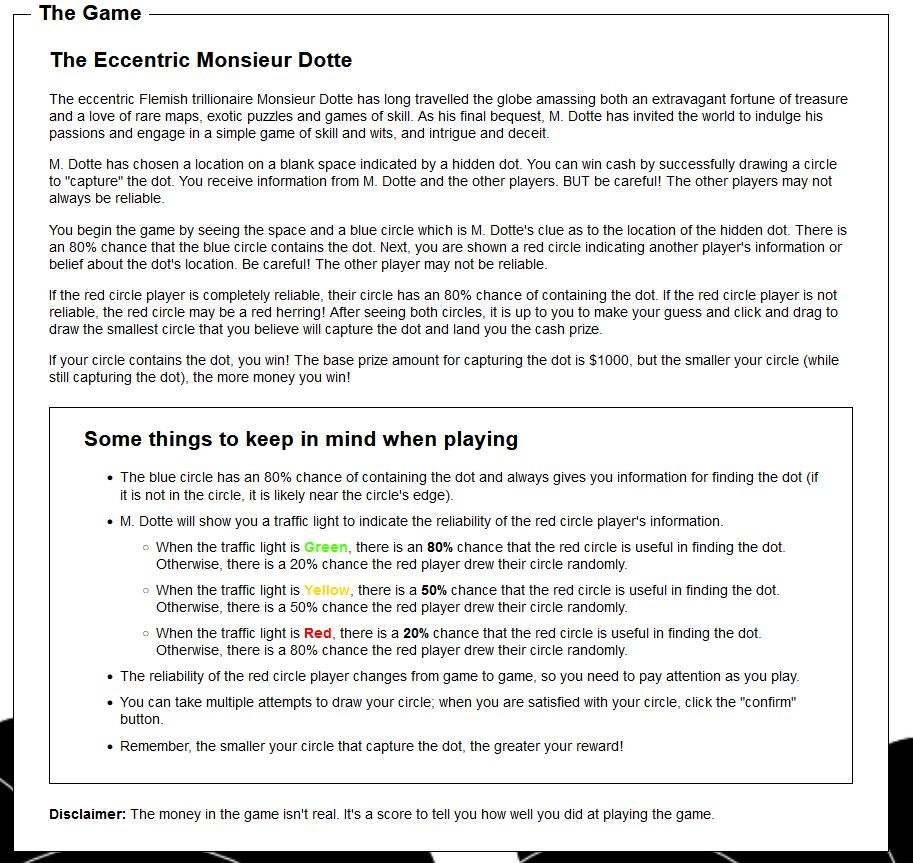

Supplement: S1 Fig — (TIF) [file pone.0275473.s001.tif]

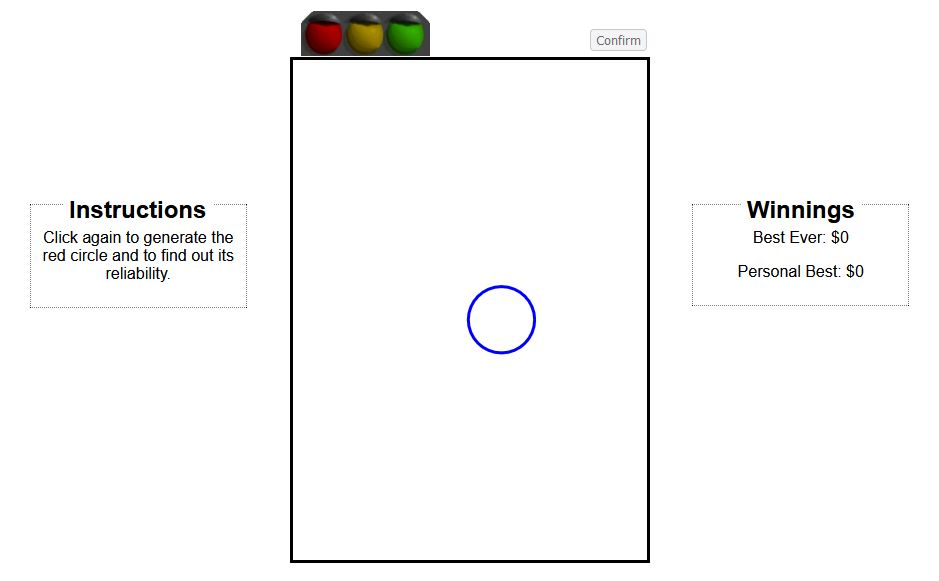

Supplement: S2 Fig — (TIF) [file pone.0275473.s002.tif]

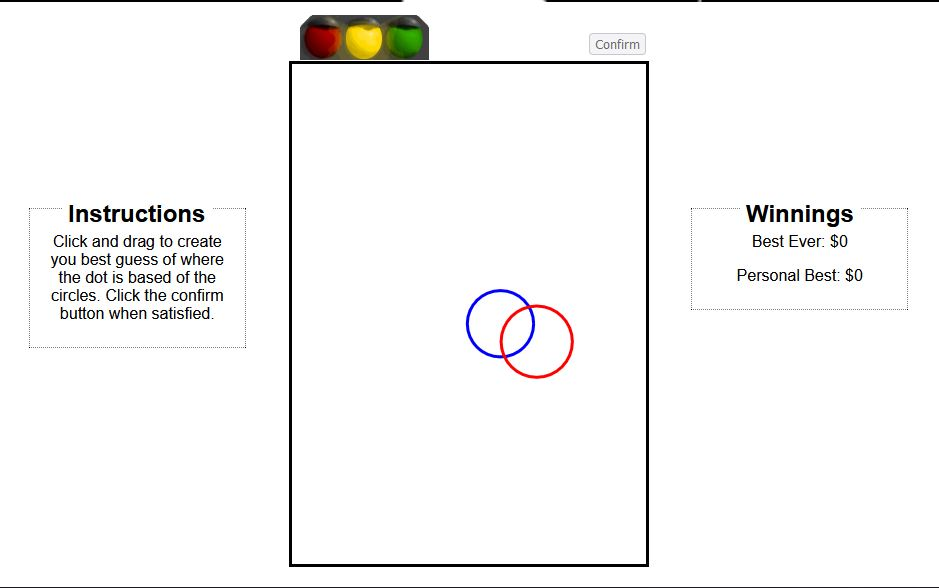

Supplement: S3 Fig — (TIF) [file pone.0275473.s003.tif]

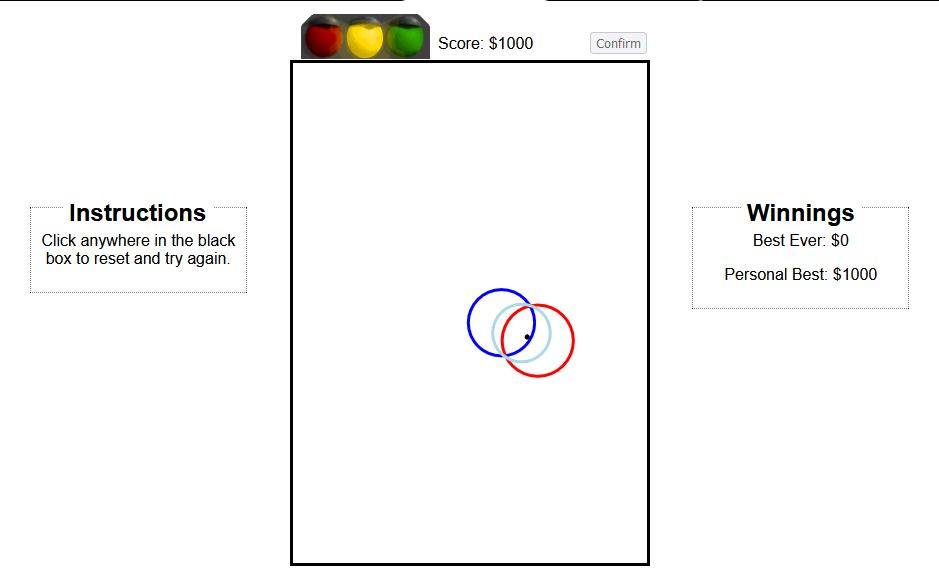

Supplement: S4 Fig — (TIF) [file pone.0275473.s004.tif]

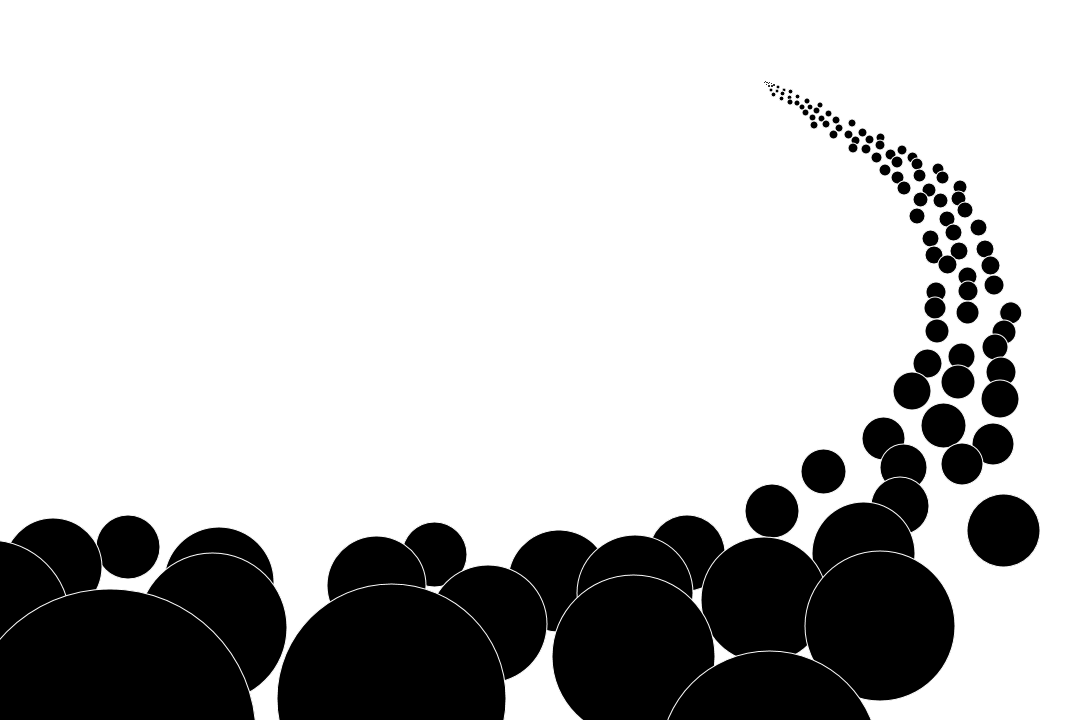

Supplement: S1 File — (ZIP) [file pone.0275473.s005.zip › img/BackgroundBG.png]

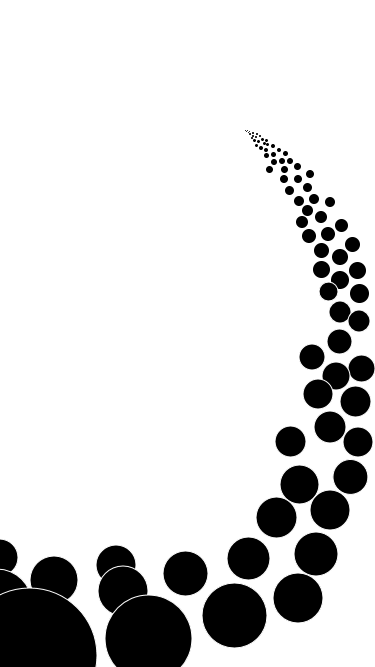

Supplement: S1 File — (ZIP) [file pone.0275473.s005.zip › img/BackgroundPhoneBG.png]

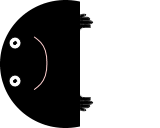

Supplement: S1 File — (ZIP) [file pone.0275473.s005.zip › img/DotTitle.png]

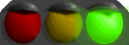

Supplement: S1 File — (ZIP) [file pone.0275473.s005.zip › img/TrafficLightGreen.png]

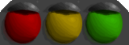

Supplement: S1 File — (ZIP) [file pone.0275473.s005.zip › img/TrafficLightInactive.png]

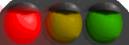

Supplement: S1 File — (ZIP) [file pone.0275473.s005.zip › img/TrafficLightRed.png]

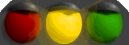

Supplement: S1 File — (ZIP) [file pone.0275473.s005.zip › img/TrafficLightYellow.png]
