# Supplementary material for: Person-to-person opinion dynamics: An empirical study using an online game
Source: PLoS One. 2022 Oct 6;17(10):e0275473. doi: 10.1371/journal.pone.0275473 (PMC9536623; doi:10.1371/journal.pone.0275473)
Supplement: S1 File — (ZIP) [file pone.0275473.s005.zip › index.html]

Finding the Dot


# Mysterious Dots and Where to find them?

# About this Website

This website hosts an experiment studying the spread of opinions in society, focusing on how people change their beliefs when presented with similar or different opinions. This experiment investigates this effect in a simple game which is explained below.

# The Game

## The Eccentric Monsieur Dotte

The eccentric Flemish trillionaire Monsieur Dotte has long travelled the globe amassing both an extravagant fortune of treasure and a love of rare maps, exotic puzzles and games of skill. As his final bequest, M. Dotte has invited the world to indulge his passions and engage in a simple game of skill and wits, and intrigue and deceit.

M. Dotte has chosen a location on a blank space indicated by a hidden dot. You can win cash by successfully drawing a circle to "capture" the dot. You receive information from M. Dotte and the other players. BUT be careful! The other players may not always be reliable.

You begin the game by seeing the space and a blue circle which is M. Dotte's clue as to the location of the hidden dot. There is an 80% chance that the blue circle contains the dot. Next, you are shown a red circle indicating another player's information or belief about the dot's location. Be careful! The other player may not be reliable.

If the red circle player is completely reliable, their circle has an 80% chance of containing the dot. If the red circle player is not reliable, the red circle may be a red herring! After seeing both circles, it is up to you to make your guess and click and drag to draw the smallest circle that you believe will capture the dot and land you the cash prize.

If your circle contains the dot, you win! The base prize amount for capturing the dot is $1000, but the smaller your circle (while still capturing the dot), the more money you win!

## Some things to keep in mind when playing

- The blue circle has an 80% chance of containing the dot and always gives you information for finding the dot (if it is not in the circle, it is likely near the circle's edge).
- M. Dotte will show you a traffic light to indicate the reliability of the red circle player's information.
  - When the traffic light is **Green**, there is an **80%** chance that the red circle is useful in finding the dot. Otherwise, there is a 20% chance the red player drew their circle randomly.
  - When the traffic light is **Yellow**, there is a **50%** chance that the red circle is useful in finding the dot. Otherwise, there is a 50% chance the red player drew their circle randomly.
  - When the traffic light is **Red**, there is a **20%** chance that the red circle is useful in finding the dot. Otherwise, there is a 80% chance the red player drew their circle randomly.- The reliability of the red circle player changes from game to game, so you need to pay attention as you play.
  - You can take multiple attempts to draw your circle; when you are satisfied with your circle, click the "confirm" button.
  - Remember, the smaller your circle that capture the dot, the greater your reward!

**Disclaimer:** The money in the game isn't real. It's a score to tell you how well you did at playing the game.

# Instructions

Press anywhere in the black box to generate the blue circle

# Winnings

Best Ever: $0

Personal Best: $0

Confirm

Your browser does not support the HTML5 canvas tag.
