# Supplementary material for: Person-to-person opinion dynamics: An empirical study using an online game
Source: PLoS One. 2022 Oct 6;17(10):e0275473. doi: 10.1371/journal.pone.0275473 (PMC9536623; doi:10.1371/journal.pone.0275473)
Supplement: S1 Appendix — (PDF) [file pone.0275473.s007.pdf]

S1 Appendix. Equations of the Martins model.

$$x_i(t+1) = p^* x_i(t) + p^* \frac{x_i(t)/\sigma_i(t) + x_j(t)/\sigma_j(t)}{1/\sigma_i(t) + 1/\sigma_j(t)},$$

$$\sigma_i^2(t+1) = \left(1 - \frac{\sigma_i^2(t)}{\sigma_j^2(t) + \sigma_i^2(t)}\right)\sigma_i^2(t) + p^*(1-p^*)\left(\frac{x_i(t) - x_j(t)}{1 + \sigma_j^2(t)/\sigma_i^2(t)}\right)^2,$$

where

$$p^* = \frac{p\phi\left(x_i(t) - x_j(t), \sqrt{\sigma_i^2(t) + \sigma_j^2(t)}\right)}{p\phi\left(x_i(t) - x_j(t), \sqrt{\sigma_i^2(t) + \sigma_j^2(t)}\right) + 1 - p},$$

and

$$\phi(\mu, \sigma) = \frac{1}{\sigma\sqrt{2\pi}} e^{\frac{-\mu^2}{2\sigma^2}}$$
